# Gender health gaps in guideline-based inpatient cardiovascular medical and nursing care and implementation strategies to reduce the gap (HeartGap): A mixed methods study protocol

**DOI:** 10.1371/journal.pone.0301732

**Published:** 2024-04-18

**Authors:** Sophia Sgraja, Judith Mollenhauer, Martina Kloepfer, Ute Seeland, Clarissa Kurscheid, Volker Amelung

**Affiliations:** 1 Institute for Epidemiology, Social Medicine and Health Systems Research, Hannover Medical School, Hanover, Germany; 2 figus–Research Institute for Health- and System Design, Cologne, Germany; 3 Institute for Gender Health, Berlin, Germany; 4 Institute for Social Medicine, Epidemiology and Health Economics, Charité, Berlin, Germany; University of Rajshahi, BANGLADESH

## Abstract

**Background:**

A growing body of evidence has demonstrated that a gender-sensitive approach to healthcare is needed in all areas of medicine. Although medical and nursing guidelines include gender-sensitive care (GSC+) recommendations, the level of implementation in health care practice is unknown. This study aims to examine the current level of implementation and acceptance of GSC+ among physicians and nurses and to identify potential gaps between guidelines and practice and barriers and facilitators of GSC+ implementation, taking the perceptions of all relevant stakeholders into account. The overarching aim is to develop holistic recommended actions to strengthen GSC+.

**Methods:**

This study has a mixed methods triangulation design. The preparation phase consisting of a literature review and a two-part (qualitative and quantitative) data analysis will be conducted in the cardiology department of 9 pilot hospitals in Berlin, North Rhine-Westphalia, Lower Saxony, Rhineland-Palatinate, Germany. 18 focus groups with clinicians and nurses as well as interviews with experts in other relevant fields will be performed. In the national roll-out phase, a questionnaire survey will be conducted with hospital clinicians (n = 382), nurses (n = 386) and patients (n = 388).

**Discussion:**

This study will provide comprehensive insights into the implementation and acceptance of GSC+ in cardiology from the perspective of doctors, nurses, patients, stakeholders and experts in relevant fields, such as policy and education. A focus will also be on the extent to which age or gender of health professionals, region and hospital type influence the implementation of GSC+. The identification of GSC+ implementation barriers and facilitators should help to improve the standard of care for cardiology patients of all genders. The outcomes from this study can be used to develop measures and recommended actions for the successful and sustainable implementation of gender-sensitive care.

**Trial registration:**

The study is registered in the German Register of Clinical Studies (DRKS) under study number DRKS00031317.

## Introduction and background

A growing body of evidence has demonstrated the need for gender-sensitive care (GSC+) in all medical specialties. Gender-sensitive care plus considers the care on “sex”—the different biological and physiological characteristics of females and males and “gender”–the socially constructed characteristics of women and men [1] and further “factors of diversity”–age, sociocultural and socioeconomic status, social support / nursing work, mental health, physical performance, religion / worldview, ethnicity and sexual identity. For myocardial infarction (MI), in-hospital lethality is still significantly higher in women than in men. Medical guidelines and nursing expert standards include recommendations for gender-sensitive care, but it is unknown how these are implemented in health care practice. It is hypothesized that there is a discrepancy between evidence-based GSC+ guidelines and expert standards and their implementation in practice. Possible reasons include lack of knowledge, acceptance, or routine use of GSC+ by health care providers. Evidence also suggests that recommendations at the medical level need to be supported by guidelines at the policy level if they are to be successfully implemented. Gender-sensitive care recommendations are intended to improve the quality of health care and to achieve gender equality and more patient-centered care [1–3].

Gender medicine has its historical roots in the USA and originated in the field of cardiology [4]. The importance of sex- and gender-sensitive medicine was recognized in Germany in the 1970s. During the second wave of the women’s movement in the 1970s and 1980s, gender equality and women’s rights to control over their bodies and reproductive rights were demanded. In the later course of the feminist movement, other neglected issues such as pathologies occurring during pregnancy and childbirth or gender differences in heart disease were increasingly addressed [5].

Initially, gender-sensitive care was approached mainly from a medical perspective without considering the nursing perspective. Even today, studies on gender-sensitive care in nursing are scarce and further research is needed [2, 3]. Patient care in hospitals is a holistic synergy between medicine and nursing. With the exception of the DNQP’s "Expert Standard for the Promotion of Urinary Continence in Nursing", German expert standards rarely include recommendations for GSC+ in nursing. The German Expert Standards, developed by the German Network for Quality Development in Nursing (Deutsches Netzwerk für Qualitätsentwicklung in der Pflege (DNQP)), aim to ensure quality in all areas of health care. They are divided into three areas: structural quality, process quality, and outcome quality. According to §113 of Social Security Code XI (SGB XI), their realization and implementation is mandatory for every nurse. Overall, gender-sensitive nursing is a young science, and only a few studies on the implementation of gender-specific nursing have been published [3, 4]. The recent Covid-19 pandemic drew increased attention to the differential impact of the disease on women and men. Support from the media promoted public awareness of the need for gender-sensitive care and research in several specialties.

Until the 2000s, clinical research predominantly utilized white men with a weight of 70 kg and a height of 180 cm as the standard [5]. The thalomide scandal in the 1960s, among others, led to an increased awareness of the need to include women in (pharmacological) research [5]. Eventually, it became a legal requirement to include women in clinical trials. Since 2014 the regulation on clinical trials (REGULATION (EU) No 536/2014 OF THE EUROPEAN PARLIAMENT AND OF THE COUNCIL of 16 April 2014 on clinical trials on medicinal products for human use, and repealing Directive 2001/20/EC) states, that the participants in the trial group should be representative of the population to be treated. In terms of research and trials, this means equal representation of women and men. However, the database of most studies is gender biased.

From a medical perspective, it is well known that appropriate consideration of gender-sensitive characteristics is indispensable for adequate patient-oriented and individualized care. Sex and gender influence the symptoms and course of disease [6]. Consequently, gender-sensitive evidence-based practice (EBP) is included in medical guidelines for several medical specialties in many different countries [7].

Cardiology is the specialty with the longest history of gender-sensitive health care research. Coronary heart disease is the most commonly diagnosed cardiovascular disease (CVD). MI is the leading cause of death in Germany [12, 13]. There is considerable evidence that the symptoms of myocardial infarction differ significantly between men and women. While men are more likely to present with left chest pain and dyspnea (the "classic" symptoms of MI), women are more likely to present with non-specific symptoms (e.g., nausea, vomiting, and dizziness) [14]. Consequently, women die more frequently in the hospital from MI than men [15, 16].

To ensure gender equality in medicine and nursing, health professionals should follow the relevant medical guidelines and nursing expert standards. In Germany, cardiologists should follow the guidelines of the European Society of Cardiology (ESC), the German Society of Cardiology–Cardiovascular Disease Research (Deutsche Gesellschaft für Kardiologie—Herz- und Kreislaufforschung e.V. (DGK)), and the Association of the Scientific Medical Societies (Arbeitsgemeinschaft der Wissenschaftlichen Medizinischen Fachgesellschaften (AWMF)). Nurses should follow the "Expert Standards" of the German Network for Quality Development in Nursing (Deutsches Netzwerk für Qualitätsentwicklung in der Pflege (DNQP)).

Numerous studies have shown differences in diagnostic and treatment outcomes between women and men [8]. Several reasons for these gender disparities and failures of implementation in health care are known [9–11]. In health care interest in the implementation of gender-sensitive care has increased [12]. According to Celik et al. [13], the successful implementation of gender sensitivity in health care practice requires the use of a multi-pronged approach that includes the professional, organizational and political insight needed to identify barriers and facilitators and to develop measures and recommendations. Other dimensions such as education, technology, sponsorship, and politics must also be taken into account.

This study of the implementation and acceptance of gender-sensitive care in cardiology in German hospitals from the perspective of clinicians, nurses, patients and other stakeholders and experts aims to provide evidence-based research findings on this topic.

### Hypotheses

The main objectives of the study HeartGap are to investigate the current level of implementation of gender-sensitive care in cardiology, to determine whether there is a discrepancy between existing evidence from guidelines and nursing expert standards and health care practice. Furthermore, the acceptance of GSC+ by clinicians and nurses will be examined. Evidence-based medical guidelines and expert standards are important tools and frameworks for the holistic implementation of medical knowledge and high-quality health care.

The following hypotheses will be evaluated in this study:

1. The translation of theoretical knowledge about gender-sensitive care (status of guidelines and expert standards) cannot be implemented sufficiently into health care practice due to a knowledge deficit (target state ≠ actual state).
2. The working conditions (staff shortages and budget pressures) are decisive factors for the low level of implementation of gender-sensitive care.
3. There is a lack of sufficient knowledge about the GSC+ requirements formulated in medical guidelines and expert standards.
4. The gender-sensitive care requirements formulated in medical guidelines and expert standards are incompatible with everyday care practice.
5. The gender-sensitive care requirements formulated in medical guidelines and expert standards do not correspond to the attitudes of health care professionals.
6. Aspects such as the age and professional experience of health care professionals and gender as well as the type of hospital and the size of the region can have an influence on the implementation of GSC+.

In summary, the HeartGap study aims to comprehensively assess the situation of GSC+ in cardiology and to investigate the correspondence between existing guidelines, expert standards and actual practice of care. The hypotheses of the study examine potential barriers, including knowledge deficits, working conditions and compatibility problems between the formulated requirements in guidelines and expert standards and daily care routines. In addition, the study recognizes the importance of contextual factors, such as nurse and physician demographics, hospital type and regional size, to the successful implementation of gender-sensitive care. Through this multi-faceted investigation, the study aims to provide valuable insights for better integration of GSC+ into cardiovascular healthcare practice.

## Methods and design

### Aim

The overarching aim of this study is to provide insight into the current level of implementation of GSC+ and guidelines for clinicians and expert standards and the willingness to implement GSC+ guidelines in practice in the German hospital sector. The study purposes to answer the research questions described below.

### Main research question

- Is the content of evidence-based gender-sensitive care guidelines for clinicians and expert standards being implemented in care practice and, if so, to what extent?

### Secondary research questions

- What are the current deficits in the implementation of GSC+ in cardiology from the perspective of clinicians, nurses and patients?
- What are the barriers and facilitators to the implementation of GSC+ in cardiology (e.g., workload, process factors, compensation systems, ignorance or uncertainty)?
- Which measures promote the implementation of GSC+ in cardiology?

### Theoretical background: Roadmap for implementation

In order to cover all relevant aspects, a theoretical framework is used for the analysis. Moise et al. [14] published a statement on behalf of the American Heart Association Council on Epidemiology and Prevention, Council on Hypertension, and Stroke Council entitled "Leveraging Implementation Science for Cardiovascular Health Equity". The authors break down the implementation of EBPs shown to promote CVD equity into four steps:

Step 1. Select and adapt EBPs.
Step 2. Identify barriers and facilitators to implementing EBPs for (CVD) equity.
Step 3. Select, use and adapt implementation strategies.
Step 4. Evaluate implementation success.

For this specific study setting, the above roadmap was adapted to create an individualized roadmap consisting of the following 5 steps:

1. Screening of medical guidelines and expert standards for gender-sensitive aspects.
2. Developing of indicators from selected gender-sensitive aspects, which are reviewed in practice by the quantitative survey.
3. Identifying barriers, facilitators and measures by a scoping review, which are relevant to the implementation of GSC+ in CVD.
4. Empirical qualitative and quantitative analysis to answer the research questions and test the hypotheses.
5. Triangulation of the data to derive recommendations for GSC+ implementation.

An evaluation of implementation success (step 4 of Moise et al.’s roadmap) will not be performed in the scope of this health care research study.

### Design

**Fig 1:**
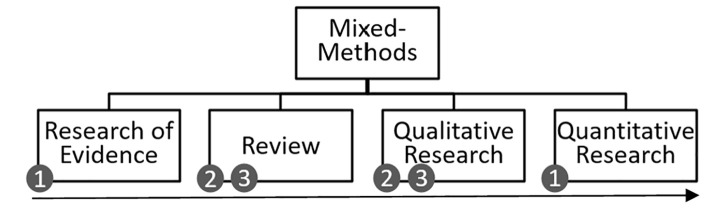
Linkage of methods and research questions. Fig 1 shows the correlation between the central research questions and the methods used to address them in this study.

This mixed methods-triangulation study includes a scoping review as well as a quantitative and qualitative analysis of the data to examine the research topic from different perspectives (Fig 1).

1. Analysis of the gap between expectations (medical guidelines and expert standards) and the current implementation by hospital physicians and nurses in practice.
2. Identification of facilitators and barriers to the implementation of gender-sensitive care.
3. Development of measures and recommended actions to improve the implementation of gender-sensitive care in practice.

In the preparation phase, the ESC guidelines and the national Association of the Scientific Medical Societies e.V.(AWMF) and (German Cardiac Society) DGK guidelines for cardiology and the DNQP expert standards for nursing were screened for GSC+ recommendations. Based on these guidelines, indicators will be developed to assess the level of implementation of gender-sensitive care. Gender-sensitive aspects of the patient pathway from admission to diagnosis, treatment and discharge will be analyzed. The implementation of these criteria will be reviewed in the course of the empirical research. The qualitative analysis will include focus group interviews conducted in 9 nationally selected pilot hospitals in separate groups of 5–7 nurses and clinicians. In addition, individual interviews will be conducted with experts from the medical and nursing professions, as well as with policymakers and other relevant stakeholders. The quantitative analysis will include questionnaire surveys that will be conducted via a nationwide roll-out including clinicians, nurses and patients.

The individual survey elements are interdependent, which means that the results of the previous part of the survey will determine the design of the following part. The elements are described in detail below.

### Setting

The HeartGap study will be conducted prospectively in the German states of Berlin, North Rhine-Westphalia, Lower Saxony, Rhineland-Palatinate. Data will be collected from these states to achieve a representative national sample including eastern and western German states, a city state, large and medium-sized cities, and a rural community. As shown in Table 1 (Attributes (number of inhabitants and grade of care) utilized as parameters to calculate stratified random sample), the study areas will be divided into three categories according to population size (large cities with more than 100,000 inhabitants, cities with 5,000 to 100,000 inhabitants, and rural communities with less than 5,000 inhabitants), and the hospitals will be divided into four categories according to level of care, defined as the number of hospital beds (teaching and research hospital with >800 beds, large hospital with > 800 beds, medium-sized hospital with 501 to 800 beds, and small hospital with ≤500 beds) (Table 1). In Germany, the classification of hospitals into care levels is based on the number of beds, which is why this classification was adopted in the course of the study. Focus groups from each level of care will be conducted in 3 regional categories. Each focus group will consist of either physicians or nurses. In the rural region, focus group interviews will be conducted in a hospital with less than 500 beds due to the lack of larger hospitals.

**Table 1:** Attributes (number of inhabitants and grade of care) utilized as parameters to calculate stratified random sample.

Level of care -------------------- Population size	Teaching / research hospital with > 800 beds	Hospital with > 800 beds	Hospital with 501–800 beds	Hospital with ≤ 500 beds
Large city (≥ 100,000 inhabitants)				
City (5,000 to 99,000 inhabitants)				
Rural community (< 5,000 inhabitants)	*Hospitals in this stratum do not exist in Germany*	*Hospitals in this stratum do not exist in Germany*	*Hospitals in this stratum do not exist in Germany*	

Table 1 illustrates how the attributes of the number of inhabitants in a region and the nursing degree of a hospital were combined as parameters for the calculation of the stratified sample.

### Recruitment

#### Qualitative survey

For the recruitment of participants in the qualitative study, the hospitals identified based on the stratification developed (as outlined in the section on Qualitative Data Collection and Analysis) are initially contacted via email. A one-page information sheet on the project will be sent out, along with a request for a digital video presentation to offer a more detailed exposition of the project. In cases where no response is received via email, follow-up telephone communication is initiated. During the video conference sessions with the hospital’s chief physicians and nursing director, a tailored recruitment strategy is devised for each institution. This strategy involves either the direct involvement of the chief physician and nursing director in approaching potential focus group participants or the engagement of the researchers associated with the project for this purpose.

The recruitment period for the qualitative survey for focus group participants (physicians and nurses) begins from March 1, 2023 to December 31, 2023 and for participants in the expert interviews (stakeholders) from March 1, 2024 to June 30, 2024. Saturation in the qualitative survey is ensured by recruiting participants based on the predefined sample (Table 1). This ensures that health care professionals from diverse hospitals at different levels of care and from different types of regions are included. If the content is not saturated, further interviews can be planned.

In addition to the focus groups, expert interviews will be conducted. Based on the dimensions identified for the scoping review, experts from areas e.g. science, politics and pharmaceuticals are recruited. At least one expert from each area will be interviewed; a total of at least 6 expert interviews are planned. This serves the purpose of recruiting experts from different areas. Expert interviews will be conducted and recruited throughout the duration of the project.

#### Quantitative survey

An a-priori sample size calculation was conducted to determine the number of cases needed. First, the number of participating clinicians, nurses and inpatients with CVD disease in cardiology and internal medicine wards was recorded. Therefore, the data of the German Hospital Quality Recording System were used. Samples were calculated using WINPEPI software according to the attribute grid (Table 1). According to our sample size calculations (Study aim: estimating a mean–stratified random sample, confidence level: 0.95, acceptable difference: 0.05 and number of strata: 9) based on the Cochran formula [15], 382 clinicians, 386 nurses and 388 cardiology patients must be included in the quantitative study during the 2-year study period (January 2023 to December 2024). WINPEPI shows the weighting per strata of how many people are to be recruited.

Utilizing pre-established connections from the qualitative investigation and the extensive network of the project consortium, facilitates the engagement with hospitals for the quantitative phase. To ensure comprehensive representation, the stratification framework developed for the qualitative study is used to include care providers from hospitals of different care levels and geographical regions. Inclusion criteria for cardiologists and nurses are, that they currently work on a cardiology ward setting, other factors such as migration are not taken into consideration in the selection of participants. In addition to the age and gender of the caregivers, the survey also asks whether the person was born in Germany in order to determine the influence of other diversity factors such as migration background. In the healthcare sector, a very high proportion of employees have a migration background, accounting for approximately 25% of all employees [16]. Therefore, the study also aims to investigate whether this aspect influences gender-sensitivity or knowledge.

For participants (physicians, nurses and patients) in the quantitative survey, the recruitment period is from October 1, 2023 to June 30, 2024. The nine pilot hospitals and further hospitals in Germany (on average four hospitals per strata are needed) are to recruit to reach the calculated sample size.

The digital questionnaires for health care professionals will be sent to the participating hospitals. The patient questionnaires will be handed out personally through research associates of the HeartGap-project and filled out around the patient´s discharge process after obtaining approval from the hospital. Patients are recruited who are being treated as inpatients for a cardiological condition.

Participating patients must be over 18 years of age and must be mentally capable of participating in the study. Project employees provide patients with assistance in completing the digital questionnaires if required.

The resulting empirical data will be accessible only to the project consortium for analysis and will be securely stored on the servers of figus GmbH and the Hannover Medical School. All participants of the qualitative and quantitative surveys will be informed about data protection and will be only allowed to participate if they sign the consent form.

### Project implementation process

**Fig 2:**
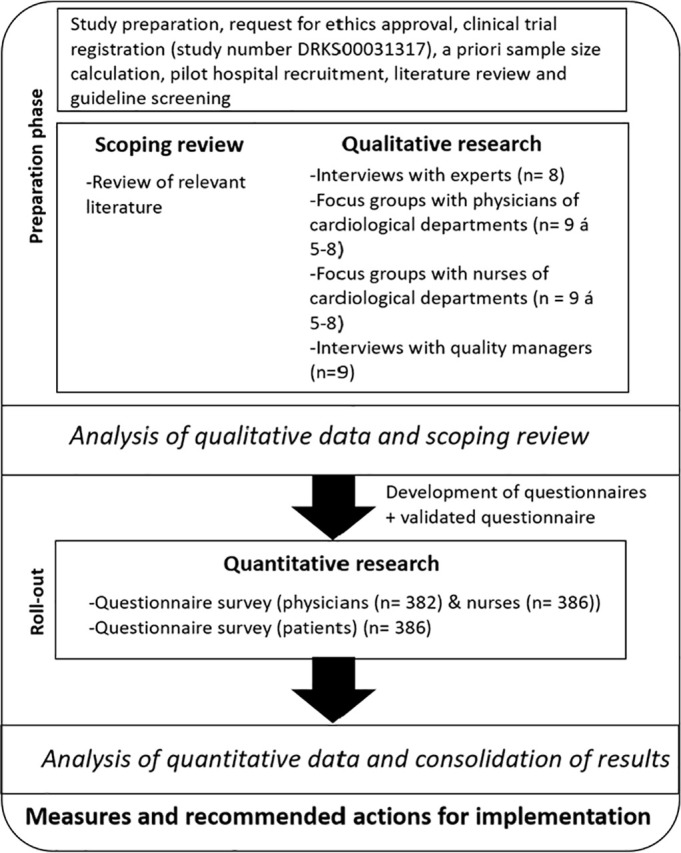
HeartGap study design. Fig 2 shows a flow chart of the project implementation process, including a description of the methodology.

The results of the literature review and the qualitative and quantitative analysis will be combined to answer the research questions (Fig 2).

### Scoping review

In preparation for the empirical research approach, a scoping literature review will be conducted taking into account the PRISMA-ScR [15]. The objective is to determine the current state of knowledge regarding the implementation of GSC+, by nurses and physicians alike. The literature research will be conducted prospectively over six months. Two researchers screen various databases such as Pubmed, Web of Science, Cinahl using search terms developed specifically for the research question. Possible key words (e.g., implementation, "gender medicine", "gender sensitivity", "gender nursing", cardiology, measure, evidence-based practice) will be combined with Boolean operators. In addition to the systematic search, a snowball system followed by a hand search will be carried out. The search will be limited to German and English publications from 2013–2023. Initially, the abstracts will be screened for eligibility by two independent researchers. Exclusion criteria will be the lack of relevance to the research questions, duplicates and publication in languages other than German or English. After abstract screening, a full-text review will be performed and articles will be included or excluded based on the defined criteria.

### Qualitative data collection and analysis

#### Focus groups

At the outset of the qualitative data collection, a total of 18 focus groups with healthcare professionals in nine pilot hospitals will be conducted. Each focus group will consist of a homogeneous group of cardiologists or nurses (four to six persons per group) from the cardiology or internal medicine department, yielding two sets of interviews per pilot hospital (one with cardiologists and one with nurses). The focus groups will be led by two researchers using semi-structured interview guidelines. Qualitative analysis provides in-depth insights into the perceptions and opinions of the respondents [17]. Therefore, exploratory questions of the following categories will be included in the interviews:

- Experiences with GSC+
- Relevant aspects of GSC+
- Implementation of GSC+ in the hospital
- Acceptance of GSC+ from a medical and nursing perspective
- Level of implementation of cardiological guidelines and expert standards in practice
- Facilitators and barriers to implementing GSC+
- Measures and recommended actions

As the aim of the study is to develop a strategy for the implementation of GSC+, the updated Consolidated Framework for Implementation Research (CFIR) will be used to consider relevant contextual factors from the outset. The CFIR indicates the complexity of implementation and consists of five domains: innovation, outer setting, inner setting, individuals and implementation process [18]. To achieve successful implementation of GSC+, these domains will be used as the basis for structuring the interview guideline and exploring barriers and facilitators.

Focus group interviews will be recorded, transcribed verbatim and pseudonymized according to ethics standards and scientific rules. The transcripts will be analyzed using MAXQDA software. A qualitative content analysis according to Mayring will be conducted [19]. Based on the transcripts, categories will be formed by deductive and inductive approaches, and new hypotheses may be developed accordingly. The results of the qualitative analysis will be considered in the later quantitative analysis.

#### Expert interviews

To obtain further objective information regarding the implementation of guidelines and expert standards in daily practice, the quality managers of the participating hospitals will also be interviewed. Interviews with care providers and expert interviews with stakeholders in the health care system are also planned. This will provide insight into the status quo and factors influencing GSC+ implementation at the different sites as well as the perception of different professions. The results of the content analysis will contribute to the development of an implementation strategy and appropriate measures and recommended actions from the perspective of health care providers, politics, education, health insurance and science.

### Quantitative data collection and analysis

Following the literature review and qualitative data analysis, questionnaires for quantitative data collection and analysis will be designed for patients and healthcare providers (clinicians and nurses). The aim is to determine the level of gender-sensitive care and to review the respondents’ knowledge and acceptance regarding the content on GSC+ in medical guidelines and expert standards (hypothesis 1). The healthcare provider questionnaire will focus on transfer of medical guidelines and expert standards in routine practice. The patient questionnaire will focus on the patient’s perception of sex and gender in the care process. The aim of the patient survey is to investigate: What needs do patients have with regard to gender-sensitive care and whether their ideas coincide with those of care providers. To collect representative data of professionals and patients, a national roll-out of the study at various hospitals in Germany is planned, and factors such as level of care and location of the hospital and socio demographic factors such as sex and age will be analyzed. The analysis is based on personal characteristics such as sex, age, education and vocational training and migration background.

In order to achieve the highest possible response rate, the survey will be conducted according to Dillman’s Total Design Method [20]. FormPro software will be used to digitize the questionnaire data. Hypotheses will be tested using descriptive and analytical methods (cluster and subgroup analysis). Statistical analysis will be performed using IBM SPSS statistical data analysis software. Validated scales will be analyzed according to the coding manual. Use of the validated Nijmegen Gender Awareness in Medicine Scale (N-GAMS) consisting of three subscales (gender sensitivity, gender stereotypes towards doctors and gender stereotypes towards patients) is also considered [21]. Other validated scales will be evaluated for appropriateness. The questionnaire will be supplemented with specially developed questions and kept as short as possible to achieve a high response rate. Quantitative data analysis will be carried out using the statistical analysis program SPSS, which is suitable for this purpose. The data will be analyzed descriptively and inferentially. Appropriate methods of statistical data analysis will be used. To test the hypotheses, absolute and relative frequencies will be calculated depending on the scale level of the variables (categorical or metric), and appropriate measures of location (arithmetic mean, median, minimum, maximum) and dispersion (standard deviation, interquartile range) will be selected and determined. Inferential statistics will be used to identify correlations. Cluster analyses and subgroup analyses will be used to compare the groups.

### Ethics approval and informed consent

The study is considered "uncritical" and has been approved by the local ethics committee of the Hannover Medical School in Hannover, Germany (approval number 1075_BO_K_2023). This ethics vote is presented to all hospitals prior to data collection. They have the option of agreeing to it or conducting their own ethics vote. The questionnaires for clinicians, nurses and patients are submitted separately to the ethics committee of Hannover Medical School before the quantitative analysis. Written informed consent will be obtained from all participants prior to enrollment. All participants will receive concise and clear information about the scope and nature of the study and how their personal data will be processed and used. Participants will be informed of their right to withdraw at any time without consequence. The study will be conducted in accordance with the principles of the Declaration of Helsinki.

## Discussion

Patients should receive medical and nursing care according to current guidelines and nursing expert standards. Therefore, this project aims to investigate the extent to which physicians and nurses implement GSC+ in guidelines in routine cardiology practice. This allows to conclude certain aspects about the gender-sensitivity of inpatient care, but additional factors need to be considered. One is the extent to which guidelines are taken into account in health care practice or used as a source of information. In addition, it will be investigated which medical guidelines are already being implemented in routine practice. It is conceivable that health care providers use reference sources other than medical guidelines and nursing expert standards. Health care providers are likely to provide GSC+ intuitively in their daily practice without explicit awareness of gender-sensitive aspects in medical guidelines or nursing expert standards. In addition to analyzing the level of implementation of GSC+, the empirical analysis will assess the gender sensitivity of physicians and nurses. In this context, a focus will be put on whether socio-demographic aspects such as age and gender can have an influence on the degree of implementation of GSC+. It is possible that younger caregivers may exhibit a heightened sensitivity toward GSC+, given the increased prominence of the topic in recent years. Conversely, experienced caregivers who have been in the profession for an extended period might be unconsciously integrating GSC+ into their practice. It may also be possible that the level of care has an influence on the implementation of GSC+, for example, better implementation is conceivable in university hospitals where there is closer proximity to research.

Another important factor is the extent to which medical guidelines and nursing reflect the current state of knowledge and evidence. As authors of medical guidelines and nursing expert standards are not obligated to include gender-sensitive aspects, their inclusion in guidelines is largely dependent on the interests of the guideline authors. In addition, guidelines often do not reflect the current state of medical evidence and it may take several years for the current state of knowledge to be reflected in the guidelines. In particular, nursing expert standards generally do not include comprehensive GSC+ provisions and tend to contain limited information on GSC+. Therefore, it may be worthwhile to include evidence from further studies on gender-sensitive care in our study.

Cardiological Guidelines and nursing expert standards contain gender-sensitive treatment of men and women but do not consider specific transgender and non-binary needs. Due to the limited number of transgender and non-binary individuals included in CVD studies, evidence-based recommendations for the gender-sensitive treatment of these individuals are lacking in medical guidelines and nursing expert standards. However, transgender and non-binary specific needs will be considered in the empirical approach of our study and if evidence in literature regarding the GSC+ of transgender and non-binary individuals is identified it may be included in our study as well.

The successful implementation of GSC+ requires a multidisciplinary approach in which different actors collaborate. Several interdependent dimensions must be considered when examining strategies to promote the implementation of GSC+. It should be given a special consideration to health policy when examining the question of GSC+ implementation, since health policy provides the framework for the implementation of GSC+ in health care practice. The results of this health care research study could contribute to the development of GSC+ policy recommendations. Currently, the state of our national gender health policy is certainly less advanced than at the international level, e.g. Canadian research institutes are leaders in gender health research (CIHR). Given the importance of GSC+ and the research deficits in Germany in this area, it is important for German policymakers to support GSC+ research in order to promote the generation of GSC+ knowledge and evidence in this specific context.

### Strengths and limitations

The strength of this health care study is in its utilization of a mixed methods approach. The combination of a literature review with methods of qualitative and quantitative analysis will enable a comprehensive examination of the topic. The triangulation approach allows for immediate consideration of preliminary results during the research process. The inclusion of the perspectives of several stakeholders provides insights into GSC+ through different lenses. The utilization of a participatory approach to GSC+ research is imperative for the development of appropriate recommended measures for implementing gender-sensitive care.

If it proves difficult to recruit enough doctors and nurses, for example, due to significant staff shortages and time pressures in the healthcare sector, changes to the study design may be necessary. Every effort will be made to use multiple channels to obtain the best possible response rates for the quantitative analysis [20]. During the course of the project, it will be necessary to react with flexibility to the recruitment situation.

The study under consideration is exclusively focused on inpatient care. Nonetheless, it is relevant to explore gender-sensitive healthcare in the outpatient sector. Particularly given the role of general practitioners or resident cardiologists as initial points of contact for patients prior to hospitalization, barring acute incidents such as myocardial infarctions. This study’s emphasis on the hospital sector is driven by considerations of thematic focus and methodological feasibility. Another aspect is the integration of nursing into the study due to a significant research gap in gender-sensitive nursing. Additionally, the study aims to assess the familiarity and implementation of GSV+ content from medical guidelines on myocardial infarction indications and nursing expert standards among healthcare providers. Considering that myocardial infarctions are predominantly treated in the inpatient sector, the setting is suitable for this examination. Moreover, the transferability of developed recommendations to other conditions will be examined. Nevertheless, it would be very interesting for future projects to also investigate gender-sensitive care in the outpatient sector. Particularly, considering the increasing ambulatory care, general practitioner practices would be a compelling research setting.

## List of abbreviations

AWMF: Arbeitsgemeinschaft der Wissenschaftlichen Medizinischen Fachgesellschaften e.V (Association of the Scientific Medical Societies)
CVD: Cardiovascular disease
DGK: Deutsche Gesellschaft für Kardiologie–Herz- und Kreislaufforschung e.V. (German Society of Cardiology–Cardiovascular Disease Research
DNQP: Deutsches Netzwerk für Qualitätsentwicklung in der Pflege (German Network for Quality Development in Nursing
DRKS: Deutsches Register Klinischer Studien (German Clinical Trials Register
EBP: Evidence-based practice; ESC: European Society of Cardiology
GSC+: Gender-sensitive care
